# Determinants of tracheostomy decannulation: an international survey

**DOI:** 10.1186/cc6802

**Published:** 2008-02-26

**Authors:** Henry Thomas Stelfox, Claudia Crimi, Lorenzo Berra, Alberto Noto, Ulrich Schmidt, Luca M Bigatello, Dean Hess

**Affiliations:** 1 Department of Critical Care Medicine, University of Calgary, Foothills Medical Centre, EG23A, 1403-29 Street NW, Calgary, AB, Canada, T2N 2T9; 2 Department of Anesthesia and Critical Care, Massachusetts General Hospital, 55 Fruit Street, Clinics 309, Boston, MA 02114, USA; 3 Department of Anesthesia and Critical Care, Policlinico Universitario 'G. Martino', University of Messina, Messina, Italy

## Abstract

**Background:**

Although tracheostomy is probably the most common surgical procedure performed on critically ill patients, it is unknown when a tracheostomy tube can be safely removed.

**Methods:**

We performed a cross-sectional survey of physicians and respiratory therapists with expertise in the management of tracheostomized patients at 118 medical centers to characterize contemporary opinions about tracheostomy decannulation practice and to define factors that influence these practices.

**Results:**

We surveyed 309 clinicians, of whom 225 responded (73%). Clinicians rated patient level of consciousness, ability to tolerate tracheostomy tube capping, cough effectiveness, and secretions as the most important factors in the decision to decannulate a patient. Decannulation failure was defined as the need to reinsert an artificial airway within 48 hours (45% of respondents) to 96 hours (20% of respondents) of tracheostomy removal, and 2% to 5% was the most frequent recommendation for an acceptable recannulation rate (44% of respondents). In clinical scenarios, clinicians who worked in chronic care facilities (30%) were less likely to recommend decannulation than clinicians who worked in weaning (47%), rehabilitation (53%), or acute care (55%) facilities (*p *= 0.015). Patients were most likely to be recommended for decannulation if they were alert and interactive (odds ratio [OR] 4.76, 95% confidence interval [CI] 3.27 to 6.90; *p *< 0.001), had a strong cough (OR 3.84, 95% CI 2.66 to 5.54; *p *< 0.001), had scant thin secretions (OR 2.23, 95% CI 1.56 to 3.19; *p *< 0.001), and required minimal supplemental oxygen (OR 2.04, 95% CI 1.45 to 2.86; *p *< 0.001).

**Conclusion:**

Patient level of consciousness, cough effectiveness, secretions, and oxygenation are important determinants of clinicians' tracheostomy decannulation opinions. Most surveyed clinicians defined decannulation failure as the need to reinsert an artificial airway within 48 to 96 hours of planned tracheostomy removal.

## Introduction

Tracheostomy is probably the most common surgical procedure performed on critically ill patients [1]. Approximately 10% of mechanically ventilated critically ill patients receive a tracheostomy to facilitate prolonged airway and ventilatory support [2-5]. The recent development of percutaneous dilational tracheostomy techniques has made tracheostomy a routine procedure commonly performed at the bedside in the intensive care unit (ICU) [6]. Prolonged tracheostomy tube placement may expose patients to an increased risk of late complications, including tracheal stenoses, bleeding, fistulas, infections, and aspiration [7-11]. Psychological implications are profound with patients experiencing reduced body image perceptions and life satisfaction [12]. Removing a tracheostomy is a fundamental step in rehabilitating a patient recovering from critical illness [13].

The frequency of tracheostomy in the management of patients receiving mechanical ventilation contrasts with the lack of evidence as to when a tracheostomy tube should be removed. It appears that the majority of critically ill tracheostomized patients who survive to ICU discharge can eventually be successfully decannulated [14]. Limited uncontrolled pilot studies [15,16] and expert guidelines [17] have proposed that decannulation be considered in patients once respiratory mechanics are adequate, mechanical ventilation is no longer needed, upper airway obstruction is resolved, airway secretions are controlled, and swallowing has been evaluated. It is unclear to what extent these publications guide clinician decision-making. To complicate matters, the long-term management of tracheostomized patients often is fragmented between different health care settings and providers because recovery from critical illness takes months [18]. Consequently, the clinicians faced with the decision whether or when to decannulate a tracheostomized patient are often not the same physicians who inserted the tracheostomy and may have limited experience with such patients. Since little is known about how clinicians make decisions to decannulate tracheostomized patients, we conducted a survey to determine the factors clinicians consider to be important in recommending decannulation and to ascertain their opinions regarding the definition of decannnulation failure.

## Materials and methods

### Study questions

We asked clinical experts around the world for their opinions regarding decannulation of tracheostomized patients. We asked four specific questions: (a) Which patient factors do clinicians rate as being important in the decision to decannulate a tracheostomized patient? (b) Which clinician and patient factors are associated with clinicians' recommendations to decannulate a tracheostomized patient in a clinical scenario? (c) To establish a definition of decannulation failure, a time frame for reinsertion of an artificial airway needs to be established. What time frame do clinicians consider for tracheostomy decannulation failure? (d) What do clinicians consider to be an acceptable rate of tracheostomy decannulation failure?

### Instrument development and testing

A survey instrument based on questionnaires by Cook and colleagues [19] and Hebert and colleagues [20] was developed to examine clinical experts' opinions regarding decannulation of tracheostomized patients. We performed a computerized search of the MEDLINE databases using the Medical Subject Headings 'tracheostomy', 'tracheotomy', and 'ventilator weaning' and the text words 'tracheostomy tube', 'artificial airway', 'extubation', and 'decannulation'. We identified all relevant articles published in the English language. We also conducted semi-structured interviews with 18 attending physicians (5 ICU physicians, 5 pulmonary medicine physicians, 5 physicians who work in a mechanical ventilation weaning unit, and 3 surgeons who perform tracheostomies), 10 respiratory therapists, 2 nurse practitioners, and 2 speech therapists to generate a list of factors contributing to the decision to decannulate a tracheostomized patient. In the initial questionnaire, we included 10 determinants of tracheostomy decannulation. The determinants initially were identified from the literature review and were selected using content experts and a Delphi method. We limited our sampling to physicians and respiratory therapists.

Based on our interviews and clinical experience, we constructed two medical and two surgical clinical case scenarios considered to be representative of the types of patients commonly treated in ICUs. Each scenario included the determinants of tracheostomy decannulation and were randomly varied as follows: age (45 versus 75 years), etiology of respiratory failure (pneumonia versus chronic obstructive lung disease), difficulty of intubation (easy versus difficult), level of consciousness (alert and interactive versus drowsy but arousable), ability to tolerate capping (tracheostomy tube capped for 24 versus 72 hours), cough effectiveness (strong versus weak cough), secretions (scant thin versus moderate thick secretions), swallowing function (enteral nutrition via a gastric tube and nothing per mouth versus enteral nutrition via gastric tube and is eating Jell-O [Kraft Foods Inc., Northfield, IL, USA] and pudding), respiratory rate (18 versus 28 breaths per minute), and oxygenation (oxygenation is 95% with an FiO_2 _[fraction of inspired oxygen] of 0.3 versus 0.5). The randomization procedure was designed to respect the logical constraints among the variables to ensure clinical consistency within the scenarios. Three scenarios were randomly selected to be included with each survey.

We asked respondents to provide basic demographic and professional data, including their experience in managing patients with tracheostomies and decannulations. For each of the potential determinants of decannulation, respondents were asked to rate the importance in their decision-making process by means of a 7-point Likert scale ranging from 1 (irrelevant) to 7 (very important). Some patients who are decannulated need to have an artificial airway reinserted and consequently respondents were asked what they considered as a time frame for decannulation failure as well as an acceptable rate of decannulation failure. Finally, a decannulation recommendation ('yes' or 'no') was requested for each of the three patient scenarios.

An assessment of the questionnaire's clarity and the completeness and realism of the scenarios was performed through piloting and semi-structured interviews with 10 ICU attending physicians and 10 respiratory therapists. Following these interviews, a few modifications were made and patient comorbidities were added as an additional determinant of tracheostomy decannulation to both the survey questions and scenarios (no significant prior comorbidities versus end-stage renal disease). The questionnaire and case scenarios are presented in Appendix A.

The instrument was formally tested in 11 clinicians (6 attending physicians and 5 respiratory therapists). Test-retest reliability was performed twice (2 weeks apart) and demonstrated an overall kappa score of 0.79 for the case scenarios. A clinical sensibility assessment using the methodology of Cook and colleagues [19] demonstrated the instrument to have good discriminability (8/11), clarity (11/11), utility (9/11), face validity (10/11), content validity (9/11), and minimal redundancy (1/11) [21].

### Survey administration

We searched the internet to identify the email addresses of the authors of tracheostomy articles identified in our initial search of the medical literature. These clinicians (*n *= 69) were sent, via email, a cover letter explaining the purpose of the study and a unique username and password that provided access to a secure web-based questionnaire (Microsoft SQL Server 2005; Microsoft Corporation, Redmond, WA, USA). Participation in the study was voluntary, and written consent was not requested but was inferred by survey completion. Reminders were sent to those clinicians who did not respond to the first mailing within 8 weeks. A second reminder was sent to those clinicians who did not respond to the first reminder within 8 weeks. A snowball sampling technique was employed and each questionnaire asked respondents to provide recommendations for additional clinical experts to survey [22]. The study was approved by the Institutional Review Board of Massachusetts General Hospital (Boston, MA, USA).

### Statistical analysis

The strategy for the primary analysis was to answer each of the four specific study questions. Survey responses were summarized using nominal (proportions), ordinal (median and interquartile range), and interval (mean and standard deviation) measures. Physician and respiratory therapist responses were compared using *t *tests, χ^2 ^tests, and Fisher exact test for outcomes with rare events. Nonparametric comparisons were performed using the Mann-Whitney and nonparametric trend tests. Logistic regression was performed to examine associations between clinician factors, patient factors, and clinicians' decannulation recommendations. Clinician factors (profession, time since graduation, primary work facility, years of tracheostomy experience, number of tracheostomy patients treated yearly, and decannulation experience) and patient factors (scenario, age, comorbidities, etiology of respiratory failure, difficulty of intubation, level of consciousness, ability to tolerate capping, cough effectiveness, secretions, swallowing function, respiratory rate, and oxygenation) were first examined using univariate analyses. Variables that were significant at a *p *value of 0.1 or less were included in the multivariable analyses. Variables were selected by means of backward stepwise regression and comparison of the regression sum of squares. Two interactions were tested: etiology of respiratory failure and patient age as well as cough effectiveness and secretions. Our patient scenario data were clustered within respondents. To account for the interdependence of these observations, we used robust estimates of variance (generalized estimating equation) [23]. Statistical analyses were performed using Stata version 9.0 (StataCorp LP, College Station, TX, USA) with two-tailed significance levels of 0.05.

## Results

### Response rate

In the end, the survey was sent to 309 clinicians (238 physicians and 71 respiratory therapists) at 118 medical centers in 10 countries (USA, Canada, Italy, Spain, France, Germany, UK, Greece, Australia, and Japan) between May and December 2006. Of the 309 clinicians who were sent the survey, 225 (73%) responded. The response rates for physicians (173/238 [73%]) and respiratory therapists (52/71 [73%]) were similar (*p *= 0.927).

### Respondent characteristics

The demographic characteristics of the respondents are summarized in Table 1. The primary specialties of practice of the physician respondents were anesthesia (17 [10%]), intensive care medicine (92 [53%]), pulmonary medicine (44 [25%]), rehabilitation medicine (1 [1%]), and surgery (19 [11%]). The majority of clinicians worked in acute care facilities, had more than 10 years of experience caring for tracheostomized patients, managed more than 50 tracheostomized patients a year, and participated in multiple tracheostomy decannulations annually.

**Table 1:** Characteristics of respondents

Characteristic	Physicians (*n *= 173)	Respiratory therapists (*n *= 52)
Time since graduation in years, mean (standard deviation)	20.3 (9.7)	18.1 (8.4)
Principal work facility		
Acute care	147 (85)	25 (48)
Weaning	17 (10)	16 (31)
Rehabilitation	7 (4)	3 (6)
Chronic care	2 (1)	8 (15)
Experience with tracheostomized patients		
<1 year	1 (1)	0 (0)
1–5 years	20 (12)	5 (10)
6–10 years	41 (24)	9 (17)
11–20 years	71 (41)	12 (23)
>20 years	40 (23)	26 (50)
Number of tracheostomized patients cared for per year		
<11 patients	6 (3)	1 (2)
11–20 patients	27 (16)	3 (6)
21–50 patients	57 (33)	11 (21)
51–100 patients	58 (34)	18 (35)
>100 patients	25 (14)	19 (36)
Number of tracheostomized patients decannulated per year		
0–1 patients	23 (13)	5 (10)
2–5 patients	32 (18)	7 (13)
6–10 patients	36 (21)	8 (15)
11–20 patients	30 (17)	10 (19)
>20 patients	52 (30)	22 (42)

Data are expressed as number (percentage) unless otherwise indicated.

### Determinants of tracheostomy decannulation

**Figure 1:**
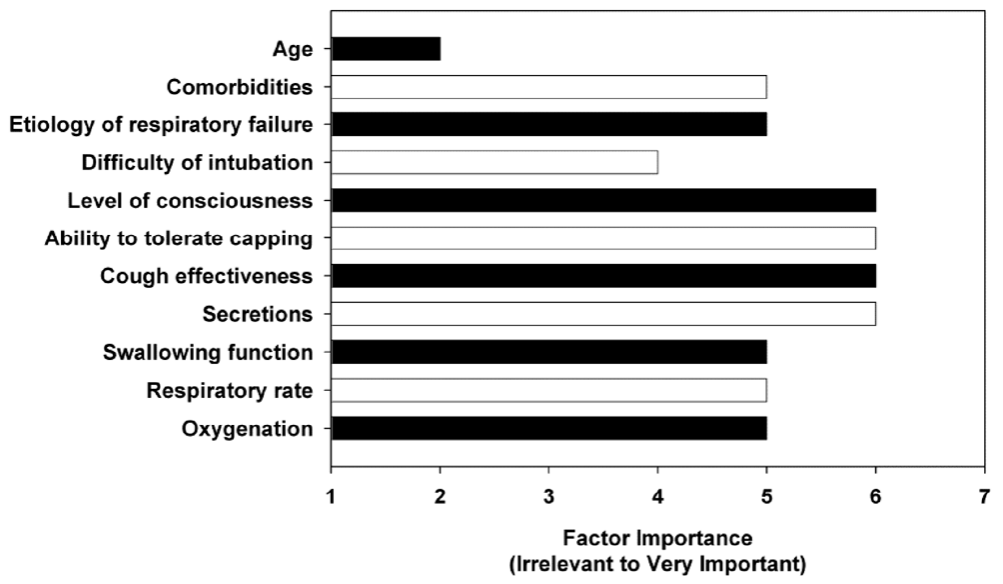
Ratings of determinants of tracheostomy decannulation. Data are expressed as median values.

Clinicians rated level of consciousness, ability to tolerate tracheostomy tube capping, cough effectiveness, and secretions as the four most important determinants in the decision to decannulate a tracheostomized patient (Figure 1). Patient comorbities, etiology of respiratory failure, swallowing function, respiratory rate, and oxygenation were judged to be of moderate importance. Patient age was the single factor that was rated as being irrevelant. Physicians rated level of consciousness (median score: 6 versus 5; *p *< 0.001) as significantly more important and ability to tolerate tracheostomy tube capping (median score: 6 versus 7; *p *< 0.001) as significantly less important than respiratory therapists did.

### Responses to scenarios

Clinicians recommended tracheostomy decannulation in 53% (355/675) of the clinical case scenarios. Decannulation recommendations did not vary according to the order (*p *= 0.220 for trend) or type (stroke, primary respiratory failure, abdominal aortic aneurysm, or trauma; *p *= 0.707 for test of proportions) of clinical scenario. Physicians (53% [272/516]) and respiratory therapists (52% [81/156]) (*p *= 0.863) were equally likely to recommend decannulation. The univariate analyses identified 8 out of 17 potential determinants of tracheostomy decannulation (Table 2). Of these variables, 5 independent factors were identified in multivariable analyses to be associated with clinicians' decannulation recommendations: 1 clinician characteristic and 4 patient scenario characteristics (Table 3). Clinicians who worked in rehabilitation (*p *= 0.063) and chronic care (*p *= 0.014) facilities were less likely to recommend decannulation. Conversely, clinicians were more likely to recommend decannulation if patients were alert and interactive (*p *< 0.001), had a strong cough (*p *< 0.001), had scant thin secretions (*p *< 0.001), and required minimal oxygen (*p *< 0.001). Patient swallowing function, though significantly associated with decannulation recommendations in univariate analyses, was not significant once examined in multivariate models that included patient level of consciousness. Two prespecified interactions were examined. There was no evidence of an interaction between etiology of respiratory failure and patient age (*p *= 0.737) or cough effectiveness and secretions (*p *= 0.102).

**Table 2:** Distribution of decannulation recommendations according to clinician and patient scenario characteristics

Characteristics	Decannulation recommendation		*P *value^a^
	Yes	No	
	(*n *= 355)	(*n *= 320)	

Clinician characteristics			
Profession			0.860
Physician	274 (77)	245 (77)	
Respiratory therapist	81 (23)	75 (23)	
Time since graduation in years (standard deviation)	18.9 (9.1)	18.4 (8.5)	0.452
Principal work facility			0.041
Acute care	282 (79)	234 (73)	
Weaning	47 (13)	52 (16)	
Rehabilitation	16 (4)	14 (4)	
Chronic care	9 (2)	21 (7)	
Experience with tracheostomized patients			0.349
0–5 years	34 (10)	44 (14)	
6–10 years	85 (24)	65 (20)	
11–20 years	134 (38)	115 (36)	
>20 years	102 (29)	96 (30)	
Number of tracheostomy patients cared for per year			0.406
0–20 patients	62 (17)	49 (15)	
21–50 patients	101 (28)	103 (32)	
51–100 patients	128 (36)	100 (31)	
>100 patients	64 (18)	68 (21)	
Number of tracheostomized patients decannulated per year			0.958
0–1 patients	45 (13)	39 (12)	
2–5 patients	65 (18)	52 (16)	
6–10 patients	68 (19)	64 (20)	
11–20 patients	63 (18)	57 (18)	
>20 patients	114 (32)	108 (34)	
Patient scenario characteristics			
Scenario			0.707
Stroke	81 (23)	85 (27)	
Primary respiratory failure	84 (24)	75 (23)	
Abdominal aortic aneurysm	98 (28)	84 (26)	
Trauma	92 (26)	76 (24)	
Age			0.103
45 years	187 (53)	147 (46)	
75 years	168 (47)	173 (54)	
Comorbidities			0.569
None	182 (51)	157 (49)	
End-stage renal disease	173 (49)	163 (51)	
Etiology of respiratory failure			0.030
Chronic obstructive pulmonary disease	192 (54)	144 (45)	
Pneumonia	163 (46)	176 (55)	
Difficulty of intubation			0.089
Easy	190 (54)	149 (47)	
Difficult	165 (46)	171 (53)	
Level of consciousness			<0.001
Alert and interactive	248 (70)	127 (40)	
Drowsy but arousable	107 (30)	193 (60)	
Ability to tolerate capping			0.626
24 hours	174 (49)	163 (51)	
72 hours	181 (51)	157 (49)	
Cough effectiveness			<0.001
Strong cough	221 (62)	117 (37)	
Weak cough	134 (38)	203 (63)	
Secretions			<0.001
Scant thin	196 (55)	129 (40)	
Moderate thick	159 (45)	191 (60)	
Swallowing function			<0.001
*Nil per os*	233 (66)	262 (82)	
Eating Jell-O and pudding	122 (34)	58 (18)	
Respiratory rate			0.129
18 breaths per minute	182 (51)	146 (46)	
28 breaths per minute	173 (49)	174 (54)	
Oxygenation, 95% saturation			
Fraction of inspired oxygen of 0.30	197 (56)	135 (42)	
Fraction of inspired oxygen of 0.50	158 (44)	185 (58)	0.001

Data are expressed as number (percentage) unless otherwise indicated. ^a^*P *value for the comparison of clinical case scenarios in which decannulation was recommended with those in which it was not. The 675 decannulation recommendations are clustered within 225 clinicians. *P *values were calculated by generalized estimating equation and robust estimates of variance.

**Table 3:** Multivariable logistic regression analysis of decannulation factors

Factors	Odds ratio (95% CI)^a^	*P *value^a^
Clinician factors		
Principal work facility		
Acute care^b^	1.00	
Weaning	0.79 (0.45, 1.40)	0.424
Rehabilitation	0.52 (0.26, 1.04)	0.063
Chronic care	0.28 (0.10, 0.77)	0.014
Patient scenario characteristics		
Level of consciousness, alert versus drowsy^b^	4.76 (3.27, 6.90)	<0.001
Cough effectiveness, strong versus weak^b^	3.84 (2.66, 5.54)	<0.001
Secretions, scant thin versus moderate thick^b^	2.23 (1.56, 3.19)	<0.001
Oxygenation, 95% saturation, FiO_2 _of 0.30 versus FiO_2 _of 0.50^b^	2.04 (1.45, 2.86)	<0.001

^a^Odds ratios with corresponding 95% confidence intervals (CIs) and *P *values were calculated by multivariable logistic regression analysis using robust estimates of variance and are for the comparison of clinical scenarios in which decannulation was recommended with those in which it was not. Odds ratios of greater than 1.0 favor clinician recommendation for tracheostomy decannulation. Odds ratios of less than 1.0 favor clinician recommendation against tracheostomy decannulation. ^b^Clinical scenarios with this factor served as the reference group. FiO_2_, fraction of inspired oxygen.

### Decannulation failure

**Figure 2:**
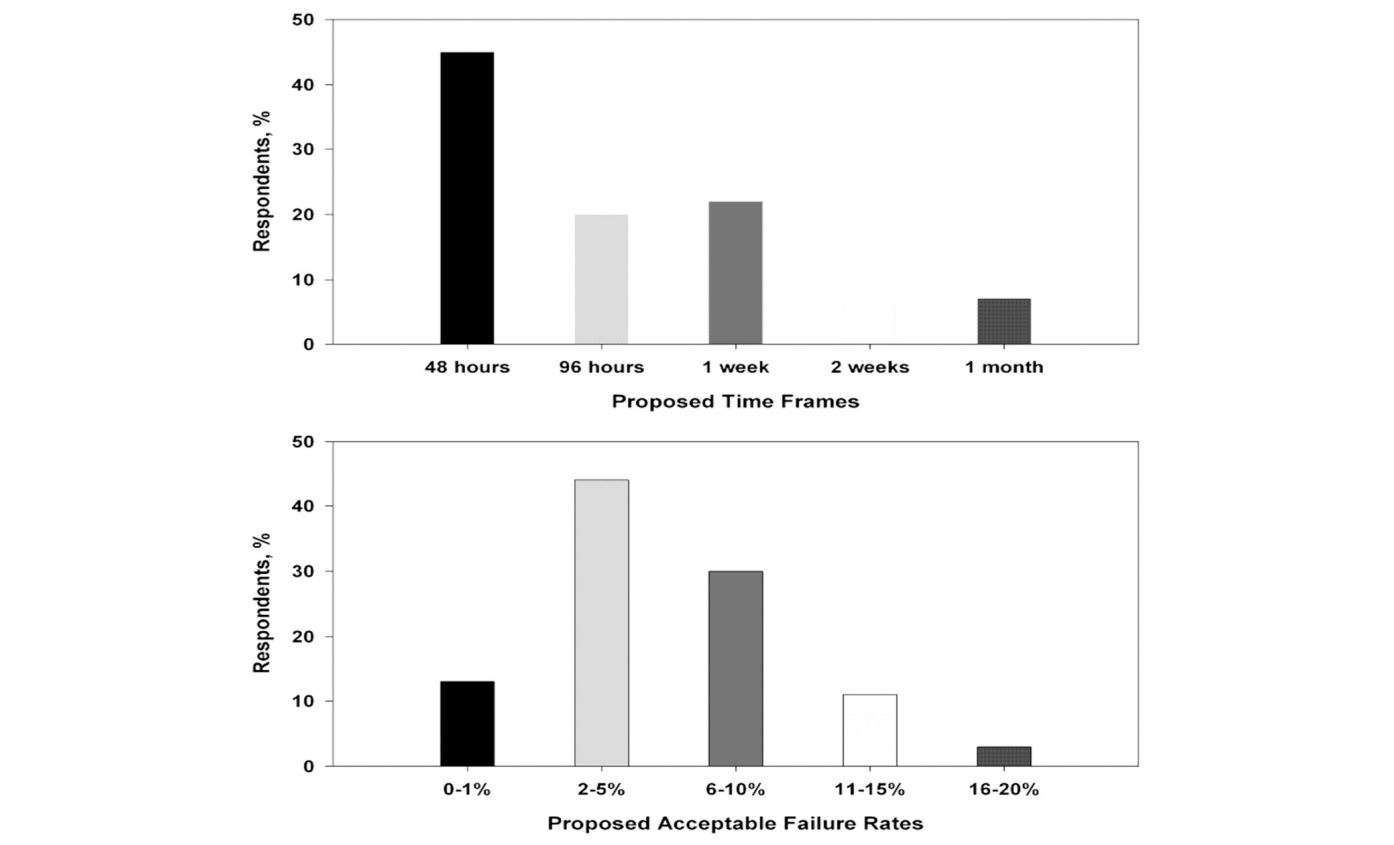
Clinician impressions of tracheostomy decannulation failure.

The distribution of clinicians' responses for describing a decannulation failure is summarized in Figure 2. The most frequent response to the question of what clinicians considered a time frame for decannulation failure was 48 hours. The median response was 96 hours. Both the median and mode for what clinicians considered an acceptable rate of failure for tracheostomy decannulation were 2% to 5%. Compared with physicians, respiratory therapists preferred shorter time frames for defining decannulation failure (median response: 96 hours versus 48 hours; *p *= 0.002 for test of proportions) but identified similar acceptable rates of decannulation failure (median response: 2% to 5% versus 2% to 5%; *p *= 0.066 for test of proportions).

## Discussion

Our study was designed to examine tracheostomy decannulation opinions at major centers around the world. The results demonstrate three major findings. First, clinicians are able to identify patient factors that they believe are important in the decision to decannulate a tracheostomized patient. Second, there is significant variability in clinicians' decannulation opinions. Third, clinicians are able to define decannulation failure and identify what they believe are acceptable rates of failure.

Clinicians are able to identify patient factors that they believe are important in the decision to decannulate a tracheostomized patient. However, there is significant variability in clinicians' opinions. For example, decannulation recommendations varied between physicians and respiratory therapists as well as between clinicians who worked at acute facilities and those who worked at chronic care facilities. Our results are consistent with a growing body of scientific literature suggesting that factors idiosyncratic to health care providers are major determinants of the medical decisions and care that patients receive [24]. Tracheostomy care, therefore, is likely to vary significantly depending on the individual clinician responsible for a patient's care. Our findings highlight the need for clinical studies in tracheostomy care to guide clinical decision-making. Clinicians indicated in our survey that, in determining whether to decannulate a tracheostomized patient, the patient's level of consciousness, ability to tolerate tracheostomy capping, cough effectiveness, secretions, and oxygenation needed to be evaluated. Although the ability to tolerate tracheostomy capping was judged to be an important determinant of tracheostomy decannulation, it did not influence clinicians' recommendations in the clinical scenarios. Previous studies and guidelines have also suggested that maximal expiratory pressure, peak cough flows, arterial blood gases, and upper airway endoscopy may be useful in the decannulation decision-making process, although these factors require special equipment and expertise and are more complicated than the simple bedside criteria employed in our study [15-17]. We propose that a patient's level of consciousness, cough effectiveness, secretions, and oxygenation be tested in a clinical trial as four simple bedside factors to consider in determining whether to decannulate a tracheostomized patient.

Clinicians understand that tracheostomy decannulation is not without risk. However, there is currently no accepted definition for decannulation failure. Extubation failure is defined by most clinicians and researchers as the need to reinstate mechanical ventilation within 24 to 72 hours of planned extubation [25-29]. The incidence of extubation failure is reported to be between 2% and 25% of extubation attempts and is associated with increased hospital mortality, prolonged ICU and hospital stays, and more frequent need for long-term acute care [27-29]. In their study of criteria for extubation and tracheostomy tube removal, Bach and Saporito [16] defined successful decannulation as 'extubation or decannulation and site closure with no consequent respiratory symptoms or blood gas deterioration for at least 2 weeks'. Ceriana and colleagues [15], in evaluating the feasibility of a decisional flowchart for weaning from tracheostomy, defined failure as the 'need to reopen the tracheotomy because of an acute episode or progressive worsening of arterial blood gases not corrected by the application of noninvasive mechanical ventilation'. The two research groups documented reinsertion of artificial airways in 35% of patients at 2 weeks and 3% of patients at 3 months, respectively [15,16]. Our data suggest that most clinicians would consider reinsertion of an artificial airway within 48 to 96 hours following planned tracheostomy removal to constitute a decannulation failure. Furthermore, clinicians appeared to consider a decannulation failure rate of 2% to 5% to be acceptable. Clinicians' preference for a lower failure rate for decannulation than extubation may be explained by the fact that tracheostomies are generally an intervention of longer duration than endotracheal intubation. Alternatively, tracheostomy may be perceived as a more invasive intervention than intubation with less tolerance for reinstrumentation. In addition, there may be a role for devices such as the Minitrach [13,15], tracheal button [17], and noninvasive mechanical ventilation [15] to serve as a bridge during the decannulation process to minimize the risk of failure, although we did not address this issue in our survey.

The results of this study need to be interpreted within the context of its limitations. First, the survey instrument used was simple and perhaps imperfect. To ensure that our instrument was economical, we were unable to explore all important aspects of tracheostomy decannulation. Second, we measured what health care workers stated they would do in response to scenarios, although we did not observe how they practice. The survey questions may have generated idealized responses rather than reflect actual practice. Finally, the snowball sampling technique efficiently identified clinical experts but provided for unequal sampling from the different jurisdictions. For example, only a small minority of respondents worked principally in a rehabilitation or chronic care facility. Nevertheless, the respondents were clinicians experienced in the management of patients with tracheostomies and represented more than 100 medical centers.

## Conclusion

Our study provides the first survey of contemporary tracheostomy decannulation practices. Our data indicate that clinicians consider a patient's level of consciousness, cough effectiveness, secretions, and oxygenation when determining whether to recommend tracheostomy removal. Clinicians define decannulation failure as the need to reinsert an artificial airway within 48 to 96 hours of planned tracheostomy removal and are willing to accept a 2% to 5% failure rate. The development of evidence-based tracheostomy guidelines will facilitate the safe and effective management of patients with tracheostomies.

## Key messages

• Patient level of consciousness, cough effectiveness, secretions, and oxygenation help clinicians determine whether to recommend tracheostomy removal.

• Decannulation failure is the need to reinsert an artificial airway within 48 to 96 hours of planned tracheostomy removal.

• Clinicians are willing to accept a 2% to 5% decannulation failure rate.

## Abbreviations

ICU = intensive care unit.

## Competing interests

The authors declare that they have no competing interests.

## Authors' contributions

HTS designed the study, analyzed and interpreted the data, drafted and revised the manuscript, and is a co-first author. CC designed the study, collected the data, drafted the manuscript, and is a co-first author. LB, US, LMB, and DH designed the study, interpreted the data, and revised the manuscript. AN developed the web-based platform, performed the randomization, interpreted the data, and revised the manuscript. All authors read and approved the final manuscript.
